# 17beta-estradiol (E2) abrogated osteolysis induced by HTLV-1 Env protein in vivo

**DOI:** 10.1186/1742-4690-8-S1-A196

**Published:** 2011-06-06

**Authors:** Yasuko Sagara, Yasuhiro Sagara, Takayuki Hara, Nobuhiro Harada, Yukiko Inoue, Nobuyo Goto, Hiroyuki Kiyokawa

**Affiliations:** 1 Japanese Red Cross Fukuoka Blood Center, Chikushino, Fukuoka, 818-8588, Japan; 2 Dept. of Early Childhood and Elementary Education, Nakamura Gakuen University, Fukuoka, 814-0198, Japan; 3 Dept. of Nutritional Sciences, Nakamura Gakuen University, Fukuoka, 814-0198, Japan; 4 Dept. of Biochemistry, Fujita Health University School of Medicine, Aichi, 470-1192, Japan

## 

HTLV-1 is the causative retrovirus of ATL. Eighty percent of ATL patients develop hypercalcemia, a severe complication resulting from bone resorption. PTHrP is known as the major pathogenic factor to hypercalcemia associated malignancies. Recently, we observed hypercalcemia in rabbits with antibody against central region of HTLV-1 Env Gp46 (gp46-197), which shows a homology with osteoprotegerin (OPG), an inhibitor to the maturation of osteoclasts. To observe the effect of gp46-197 synthetic peptide on osteogenesis, the peptide was intraperitoneally administered to mice in the presence or absence of co-administration of E2. Seven days later, serum E2 level and bone mineral density (BMD) were measured. Both of males and females exhibited a clear reduction in E2 and BMD by the gp46-197 peptide. Histomorphological analyses revealed that the peptide induced the loss of Ob.S/BS and increase in ES/BS, N.Oc/B.Pm, and Oc.S/BS. These results suggest that the structural mimicry of gp46-197 inhibits the OPG function and promote the maturation and stimulation of osteoclasts in vivo. Co-administration of E2 abrogated the adverse effects by gp46-197. This is a novel mechanism of osteolysis directly induced by HTLV-1 structural protein. We presume the therapeutic potential of E2 for the treatment of hypercalcemia in ATL patients.

